# Homogeneity evaluation of mesenchymal stem cells based on electrotaxis analysis

**DOI:** 10.1038/s41598-017-09543-0

**Published:** 2017-08-29

**Authors:** Min Sung Kim, Mi Hee Lee, Byeong-Ju Kwon, Dohyun Kim, Min-Ah Koo, Gyeung Mi Seon, Jong-Chul Park

**Affiliations:** 1 Cellbiocontrol Laboratory, Department of Medical Engineering, Yonsei University College of Medicine, Seoul, 120-752 Korea; 2 Brain Korea 21 PLUS Project for Medical Science, Yonsei University College of Medicine, Seoul, 120-752 Korea

## Abstract

Stem cell therapy that can restore function to damaged tissue, avoid host rejection and reduce inflammation throughout body without use of immunosuppressive drugs. The established methods were used to identify and to isolate specific stem cell markers by FACS or by immunomagnetic cell separation. The procedures for distinguishing population of stem cells took a time and needed many preparations. Here we suggest an electrotaxis analysis as a new method to evaluate the homogeneity of mesenchymal stem cells which can observe the stem cell population in culture condition and wide use to various types of stem cells. Human mesenchymal stem cell, adipose derived stem cell, tonsil derived stem cell and osteogenic differentiated cells migrated toward anode but the migration speed of differentiated cells was significantly decreased versus that of stem cells. In mixture of stem cells and differentiated cells condition, we identified that the ratio of stem cell versus differentiated cell was matched with the homogeneity evaluation data of stem cells based on electrotaxis analysis. As a result, our evaluation tool has the possibility of the wide use to stem cell homogeneity evaluation and might be used as the stem cell quality control during stem cell culture without any additional antibodies.

## Introduction

In recent years there have been tremendous studies in the stem cell therapy, because it has some advantages which can restore function to damaged or diseased tissue, avoid host rejection and reduce inflammation throughout the body without the use of immunosuppressive drugs^1^. Specifically adult stem cells, multipotent cells with the capacity to promote angiogenesis, differentiate to produce multiple types of connective tissue and down-regulate an inflammatory response are the focus of a multitude of clinical studies currently under way. The stem cells are being explored to regenerate damaged tissue and treat inflammation, resulting from cardiovascular disease and myocardial infarction, brain and spinal cord injury, stroke, diabetes, cartilage and bone injury^2^. In stem cell therapy, the differentiated cell ratio is very important because there is a risk to form a tumor when the undifferentiated cells were implanted into body^3^. However the current differentiation protocols of human stem cells are not able to synchronize the birth and development of cell populations to the extent seen in normal development, and consequently cells at different stages of maturation are present in such cultures, causing a cellular heterogeneity that impedes experimental and clinical utility^4–7^. To solve these problems, the homogeneity of stem cells needed to be identified before the application and the evaluation technique of stem cell homogeneity is strongly demanded.

Flow cytometric analysis and fluorescence-activated cell sorting (FACS) provide separation of cellular populations based on fluorescent labeling, for example according to surface antigens^8, 9^. After such work has been accomplished, defined combinations of surface markers can be used to identify and to isolate specific stem cell markers by FACS or by immunomagnetic cell separation (MACS)^10^. Such stem cell selection procedures and marker sets will enable the analysis, characterization, and separation of distinct subpopulations of stem cells for basic studies of stem cell biology, development, and potential therapeutic application. However these evaluation techniques of stem cells took a time and needed many preparations, so new stem cell selection methods are needed to realize the possible scientific and clinical benefits of using human stem cells.

The cell migration is influenced by the direct electric current and this phenomenon is called ‘Electrotaxis’^11^. The direction or migration speed of cells was influenced by the direct current and the electrotaxis was specific to the cell types. Because of this specificity, electrotaxis is very helpful to study the cell migration characteristics and also this electrotaxis could be a characteristic of each cell. Here we suggest an electrotaxis analysis as a new method to evaluate the homogeneity of stem cells.

## Materials and Methods

### Cell Culture

Adipose derived stem cell (ADSC, Lonza, Basel, Switzerland) were cultured in adipose derived stem cell growth medium (ADSCGM, Lonza). Human mesenchymal stem cells (hMSC, Lonza, Basel, Switzerland) were cultured in mesenchymal stem cell growth medium (MSCGM, Lonza). Tonsil mesenchymal stem cells (TMSC) were provided by Dr. Jo in Ewha woman’s university (Seoul, Korea) and cultured in DMEM (Welgene, Seoul, Korea)^12^. Cells were incubated at 37 °C in a 5% CO_2_ atmosphere. ADSC, hMSC and TMSC passages between 3 and 5 were used in all experiments.

### Osteogenic differentiation

Osteogenic differentiation (OsD) of stem cells was performed at defined passages 3–5. To promote osteogenic differentiation, the cells were seeded at a density of 3.1 × 10^3^ cells per cm^2^ into 75 T flask and cultured in ADSCGM for ADSC, MSCGM for hMSC and DMEM for TMSC until they reached 70–80% confluence. As soon as subconfluence was reached, osteogenic differentiation of the cells was induced by feeding them for 2 weeks, twice a week with osteogenic induction medium (Lonza, Basel, Switzerland) for ADSC and hMSC. DMEM with 50 µg/ml ascorbic acid, 10 mM B-glycerophosphate, 10 nM dexamethasone was used for TMSC osteogenic differentiation^12^.

### Preperation of stem cell vs. OsD cell mixture

The stem cells were cultured in osteogenic induction medium to make the OsD cells. The stem cells and OsD cells were mixed at ratio 3:7, 5:5, 7:3. OsD 3, 7, 14d cells means that the cells which were culture in osteogenic induction medium for 3, 7, 14 days. To make 3:7 ratio of mixture, for example stem cells and OsD cells were detached by trypsin and 3 × 10^3^ stem cells, 7 × 10^3^ OsD cells were injected to 1 ml of suspension.

### Electrotaxis on stem cells

To apply a direct electric current to stem cells and osteogenic differentiated cells, we used a customized agar-salt electrotaxis incubator and chamber system^13^. The electrotaxis chamber and incubator system consisted of the incubator system and electrotaxis chamber. The incubator system was installed with a microscope to observe live cells and the electrotaxis chamber applies a direct electric current to the cells. The incubator which maintains the proper growth environment (CO_2_ 5%, 37 °C) is regulated by a temperature and gas composition-controlling program (CCP ver. 3.8, Live Cell Instrument, Seoul, Korea). A cell seeded slide glass was mounted on the chamber bottom, and the chamber top and silicon gasket were placed on top of the slide. At the end of the chamber top was connected with 2% agar-salt bridge. To sterilize the chamber, the chamber was dipped in 70% ethanol and washed three times with distilled water (DW). The cells were seeded at 3 × 10^3^ cells density on the slide glass with silicon O-ring (inner diameter 16 mm) and incubated for 16~24 h in the CO_2_ incubator.

### Analysis of cell migration

The customized agar-salt electrotaxis chamber was placed on the microscope stage. The cell images were recorded every 5 minutes using a charge-coupled device (CCD) camera (Electric Biomedical Co. Ltd., Osaka, Japan) attached to an inverted microscope (Olympus Optical Co. Ktd., Tokyo, Japan). The images were saved to the computer by using the Tomoro image capture program and images were stored as JPEG files. These images were moved into ImageJ program (ImageJ 1.37v by W. Rusband, National Institutes of Health, Bethesda, MD, USA). Image analysis was carried out by the manual tracking and chemotaxis tool plug-in (v. 1.01, distributed by ibidi GmbH, Munchen, Germany). We obtained XY coordinates using manual tracking. The data were then imported into the chemotaxis plug-in. This tool was used to compute cell migration speed and x directedness both of which were used to plot the cell migration pathway. The migration speed, referred to as the migration speed, indicates how fast cells move in response to the stimulation, calculated using the total length of the migration path divided by the total observation time^11, 14^. The x directedness of the cell was defined as the straight-line distance along the x-axis between the start position and the end position of the cell divided by the length of straight-line between the start position and the end position of the cell. A cell moving directly to the right (direction of the electric current) would have an x directedness of 1, and a cell moving directly to the left (opposite direction of the electric current) would have an x directedness of −1. The directedness value close to 0 represents random cell movement. Therefore, the average directedness of a population of cells gives an objective quantification about the direction of the migrated cells.

### Immunofluorescence Observation

Stem cells and osteogenic differentiation were observed by immunofluorescence staining. Stem cells and osteogenic differentiated cells were fixed with 4% formaldehyde for 30 min at room temperature and then washed 3 times with PBS. Cells were permeabilized with 0.1% Triton X-100 in PBS for 5 min at room temperature and rinsed 3 times with PBS. 3% bovine serum albumin was treated for 30 min at room temperature and cells were incubated with a RUNX2 primary antibody (dilution 1:100, BD Transduction Laboratories TM, BD Biosciences, CA, USA) overnight at 4 °C. Cells were then washed at least 3 times with PBS and treated with CD-105 primary antibody (dilution 1:100, BD Transduction Laboratories TM) for 1 h. After 3 times washing with PBS, Alexa (488) (5 U/ml, Invitrogen, Carlsbad, CA, USA) for the RUNX2 and goat anti-mouse IgG conjugated with Texas Red (dilution 1:50, Santa Cruz, CA, USA) for CD105, treated for 1 h at room temperature in the dark. After PBS washing, cells were treated with Hoechst #33258 (Sigma, St. Louis, MO, USA) for 5 min at room temperature in the dark. The cell seeded slide glass was then mounted on the fluorescence-inverted microscope (LSM700, Carl Zeiss, New York, USA) and observed.

### Homogeneity evaluation of stem cells by electrotaxis analysis

To evaluate the stem cell homogeneity, we used migration speed histograms and electrotaxis analysis. The cell migration data were analyzed using SAS software (version 9.4, SAS Inc., Cary, NC, USA). The threshold of stem cell migration speed vs. osteogenic differentiated cells at day 3, 7, 14 was determined by optimal cut-off value (Youden’s J index)^15^. This index can be defined as J = max {sensitivity of all possible threshold values (Se(c)) + specificity of all possible threshold values (Sp(c)) − 1} and ranges between 0 and 1. Complete separation of the distributions of the marker values for the diseased and healthy populations results in J = 1 whereas complete overlap gives J = 0^15^. J provides a criterion for choosing the “optimal” threshold value (c*), the threshold value for which Se(c) + Sp(c) − 1 is maximized^16^.

### Statistical Analysis

Data were reported as means ± standard error of the mean (SEM). Means were compared using one-way analyses of Student’s t-tests. A value of p < 0.05 is considered statistically significant.

## Results

### Osteogenic differentiation of stem cells

**Figure 1:**
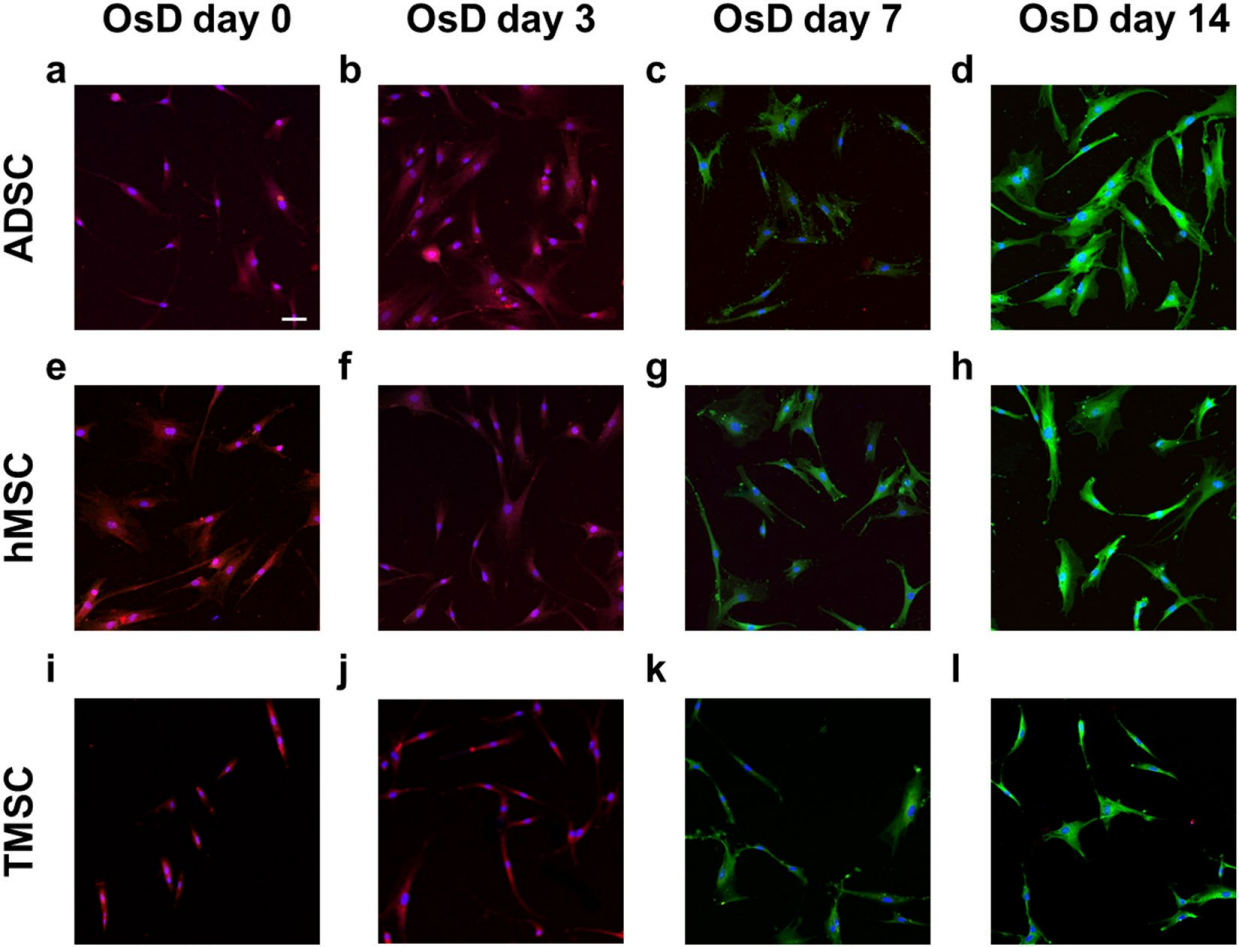
The immunofluorescence images of stem cells and osteogenic differentiated stem cells. The immunofluorescence images of ADSC osteogenic differentiation at (**a**) day 0, (**b**) day 3, (**c**) day 7, (**d**) day 14. The immunofluorescence images of hMSC osteogenic differentiation at (**e**) day 0, (**f**) day 3, (**g**) day 7, (**h**) day 14. The immunofluorescence images of TMSC osteogenic differentiation at (**i**) day 0, (**j**) day 3, (**k**) day 7, (**l**) day 14. The nuclei were stained with Hoechst #33258 (blue), the RUNX2 (osteogenic differentiation marker) was stained with Alexa (488) (green), and CD-105 (ADSC marker) was stained with Texas Red conjugated antibody (red). Scale bar = 100 μm.

Mesenchymal stem cells (MSC) are identified as a multipotent cell population in the adult organism able to be induced to express adipogenic, osteogenic and chondrogenic markers^17–21^. Osteogenic differentiation was confirmed by the detection of stem cell marker (CD-105) and osteogenic differentiation marker (RUNX2) by immunofluorescence staining. Figure 1 shows the fluorescence images of ADSC, hMSC, TMSC and osteogenic differentiated cells. CD-105 was strongly detected at OsD day 0 and day 3 but RUNX2 was not expressed at the same period. RUNX2 was detected at day 7 while the intensity of CD-105 decreased at OsD day 7. At day 14 of OsD, the intensity of RUNX2 was increased but CD-105 was not detected. These results suggested that ADSC, hMSC and TMSC were fully differentiated at day 14 by using the osteogenic differentiation media. The effect of electric current on the osteogenic differentiation was also identified. S1 showed that CD-105 of ADSC which were treated by 1000 µA of electric current was detected at 7, 14, 21 days after electric treatment, however RUNX2 was not observed. In hMSC and TMSC case, CD-105 and RUNX2 showed same results as ADSC (S2, S3).

### Electric current induced directional migration of stem cells and osteogenic differentiated cells

**Figure 2:**
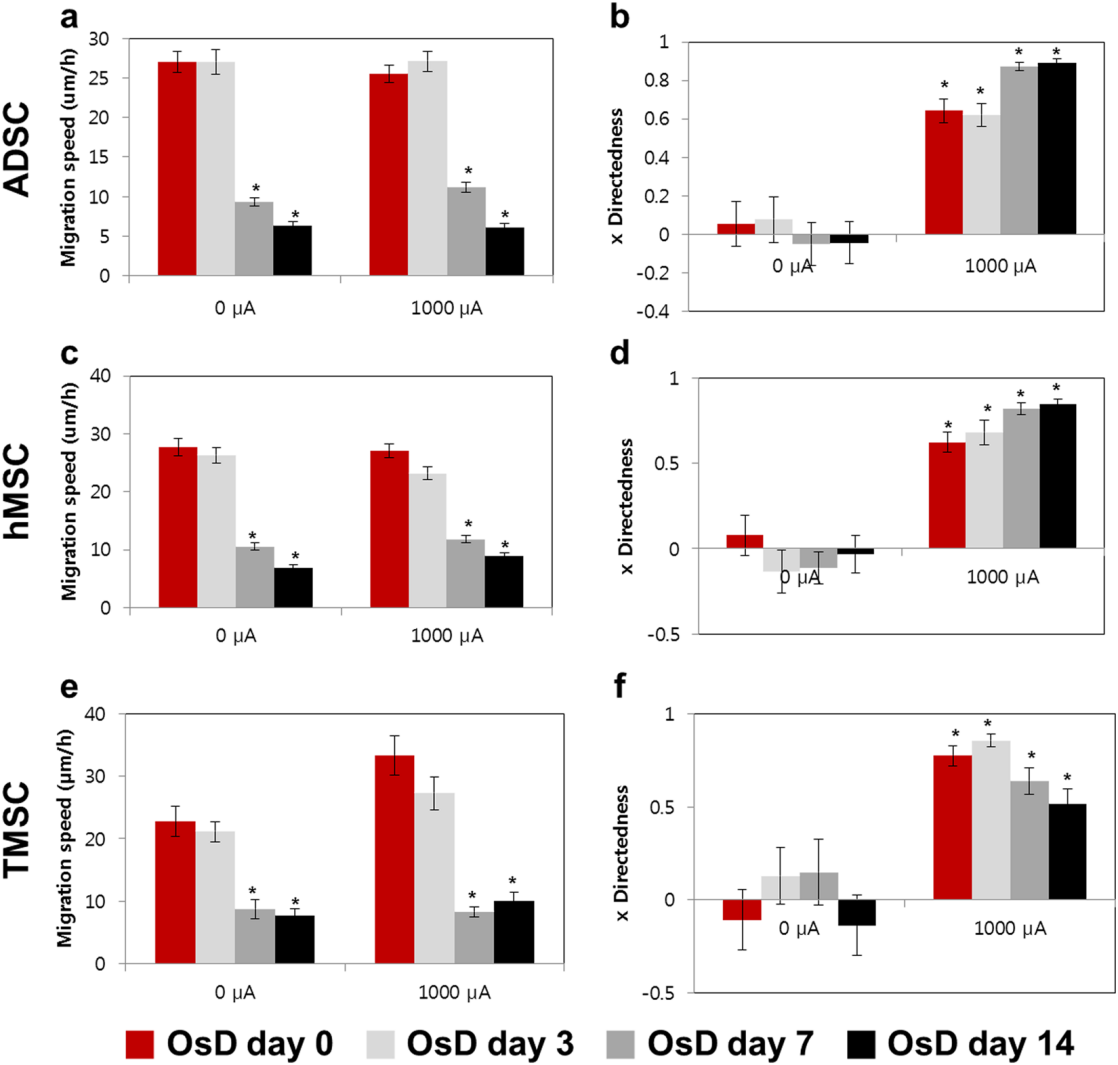
Migration data of stem cells and osteogenic differentiated cells. (**a**) Migration speed of ADSC and osteogenic differentiated cells at day 0, 3, 7, 14 with 0, 1000 μA for 3 h. (**b**) x directedness of ADSC and osteogenic differentiated cells at day 0, 3, 7, 14 with 0, 1000 μA for 3 h. (**c**) Migration speed of hMSCand osteogenic differentiated cells at day 0, 3, 7, 14 with 0, 1000 μA for 3 h. (**d**) x directedness of hMSC and osteogenic differentiated cells at day 0, 3, 7, 14 with 0, 1000 μA for 3 h. (**e**) Migration speed of TMSC and osteogenic differentiated cells at day 0, 3, 7, 14 with 0, 1000 μA for 3 h. (**f**) x directedness of TMSC and osteogenic differentiated cells at day 0, 3, 7, 14 with 0, 1000 μA for 3 h. *p < 0.05 compared to the 0 d with 0 μA for 3 h.

To analyze the change of migration pattern of stem cells and osteogenic differentiated cells, we used electrotaxis. Figure 2 showed the cell tracking data of ADSC, hMSC, TMSC and osteogenic differentiated cells at OsD day 3, 7, 14 with 0, 1000 μA for 3 h. ADSC, hMSC and TMSC showed no directional migration with no electric current, however moved to the anode when the 1000 μA of electric current was applied to the cells. The migration speed was significantly decreased at OsD day 7 and 14 compared to the day 0 (Fig. 2 (a,c,e)). The migration speed was not affected by the electric current. However, x directedness of electric current treated group was significantly increased compared to day 0 with 0 μA (Fig. 2(b,d,f)). These results indicated that the more MSCs osteogenic differentiated, the more migration speed decreased but the direction of MSC migration by electrotaxis was not affected. Therefore we could use the migration speed as an independent factor of electrotaxis analysis.

### Effect of electrotaxis on cell viability

**Figure 3:**
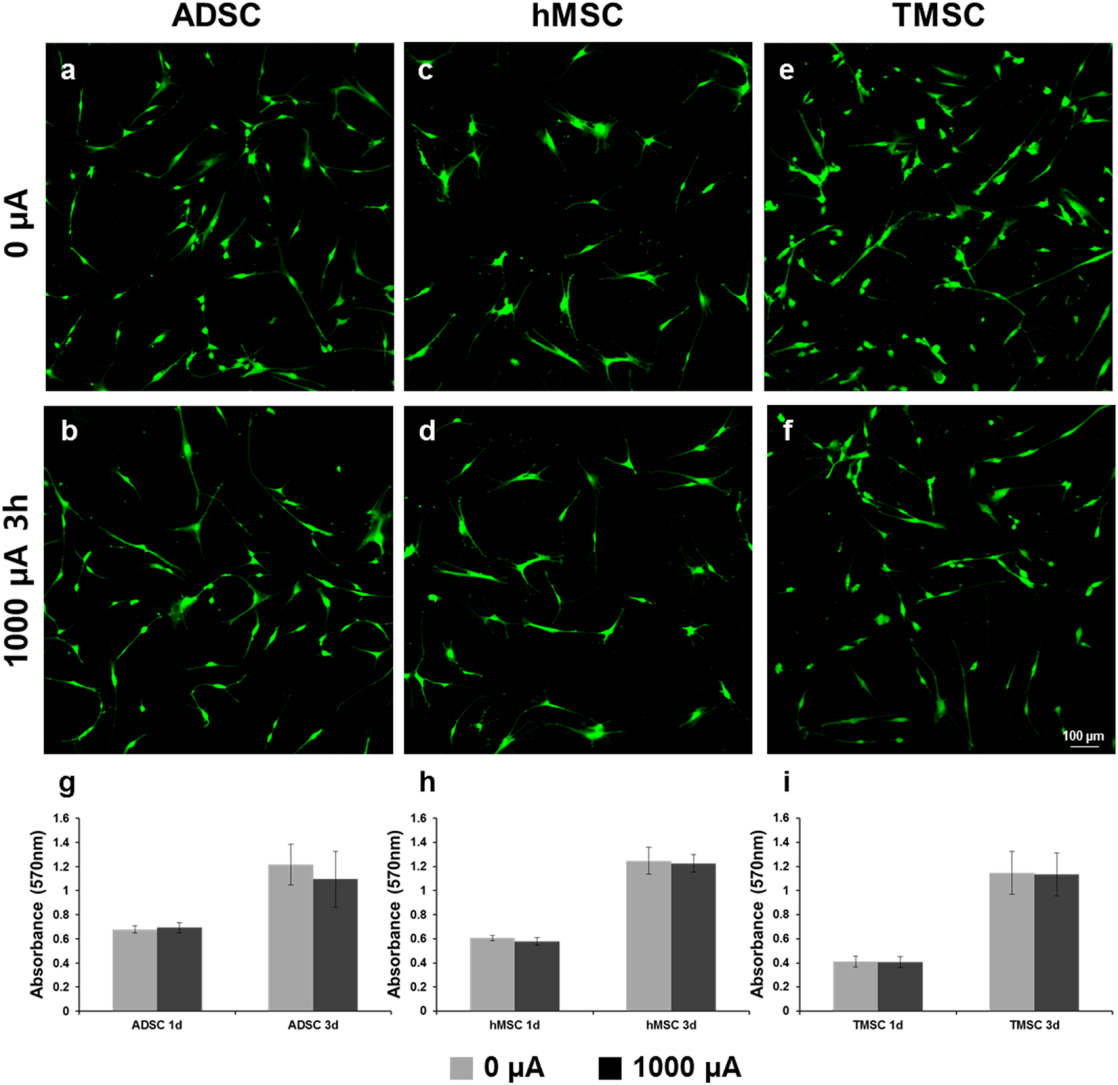
The effect of direct electric current on the cell viability. The LIVE/DEAD assay images of (**a**) ADSC under 0 µA, (**b**) ADSC under 1000 µA for 3 h, (**c**) hMSC under 0 µA, (**d**) hMSC under 1000 µA for 3 h, (**e**) TMSC under 0 µA, (**f**) TMSC under 1000 µA for 3 h (live cells: green, Calcein AM/dead cells: red, EthD-1). Scale bar = 100 μm. The MTT assay of (**g**) ADSC, (**h**) hMSC, (**i**) TMSC for 1, 3d culture after 0 or 1000 µA treatment for 3 h.

To evaluate the effect of electrotaxis on the stem cell viability, Live/Dead assay and MTT assay were performed. The Live/Dead assay was carried out right after the 1000 µA of electric current. Only green dyed cells which mean the live cells were observed in ADSC, hMSC and TMSC case, and there were no dead cells (Fig. 3(a)~(f)). In MTT assay data at 1d and 3d, there were no differences between 0 µA and 1000 µA of electric treatment on stem cells (Fig. 3(g)~(i)). These results showed that stem cell viability was not affected by 1000 µA of electric current.

### Evaluation of stem cell homogeneity by electrotaxis analysis

**Figure 4:**
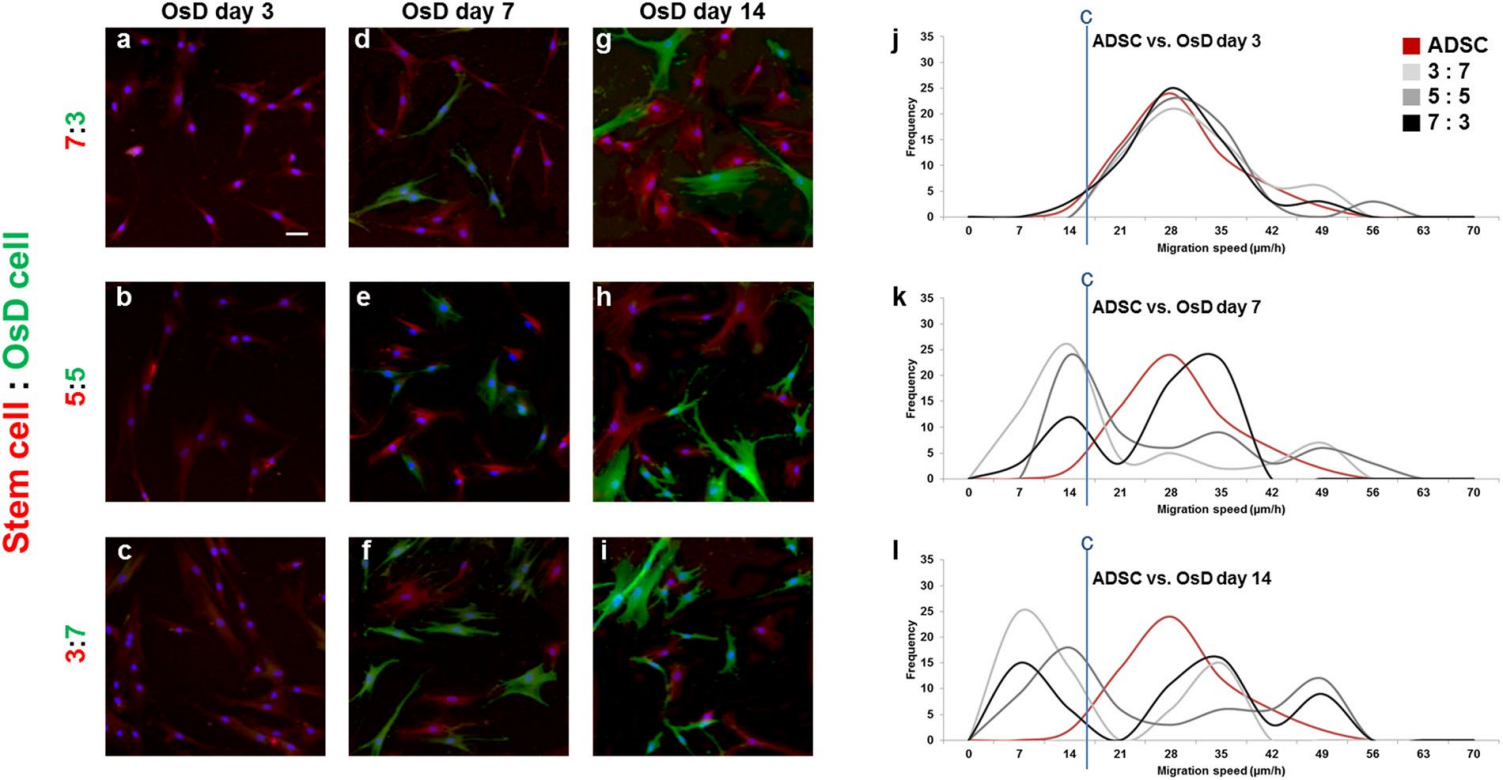
Mixture of ADSC and osteogenic differentiated cells at different OsD days. The immunofluorescence images of ADSC and osteogenic differentiated cells mixture ratio of (**a**) 7:3, (**b**) 5:5, (**c**) 3:7 at day 3, (**d**) 7:3, (**e**) 5:5, (**f**) 3:7 at day 7, and (**g**) 7:3, (**h**) 5:5, (**i**) 3:7 at day 14. The histogram of ADSC and osteogenic differentiated cell mixture using electrotaxis analysis and Youden index under 1000 µA at (**g**) day 3, (**h**) day 7, (**i**) day 14. Cut point of ADSC vs. osteogenic differentiated cells was 18.71 μm/h. Scale bar = 100 μm.

**Figure 5:**
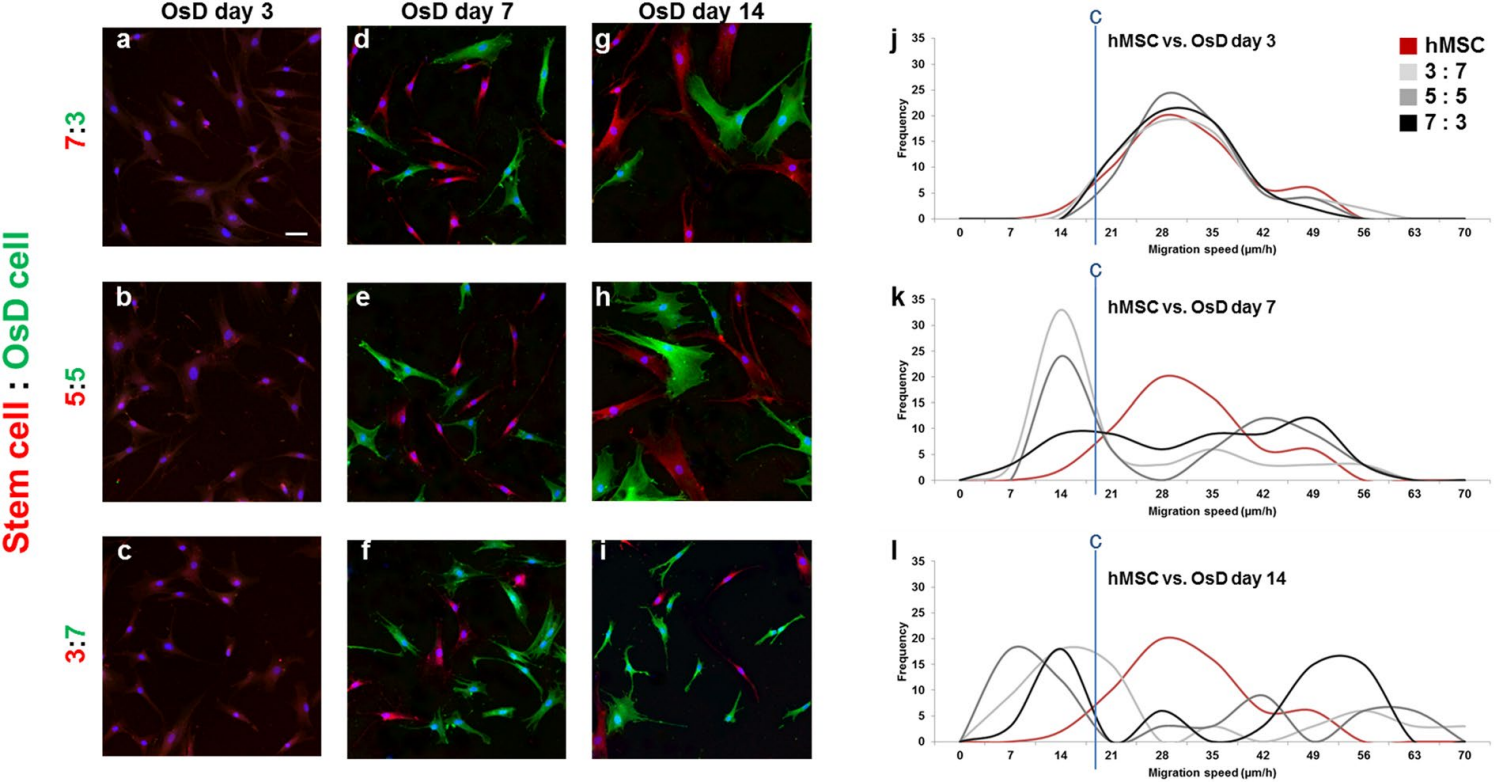
Mixture of hMSC and osteogenic differentiated cells at different OsD days. The immunofluorescence images of hMSC and osteogenic differentiated cells mixture ratio of (**a**) 7:3, (**b**) 5:5, (**c**) 3:7 at day 3, (**d**) 7:3, (**e**) 5:5, (**f**) 3:7 at day 7, and (**g**) 7:3, (**h**) 5:5, (**i**) 3:7 at day 14. The histogram of hMSC and osteogenic differentiated cell mixture using electrotaxis analysis and Youden index under 1000 µA at (**g**) day 3, (**h**) day 7, (**i**) day 14. Cut point of hMSC vs. osteogenic differentiated cells was 18.73 μm/h. Scale bar = 100 μm.

**Figure 6:**
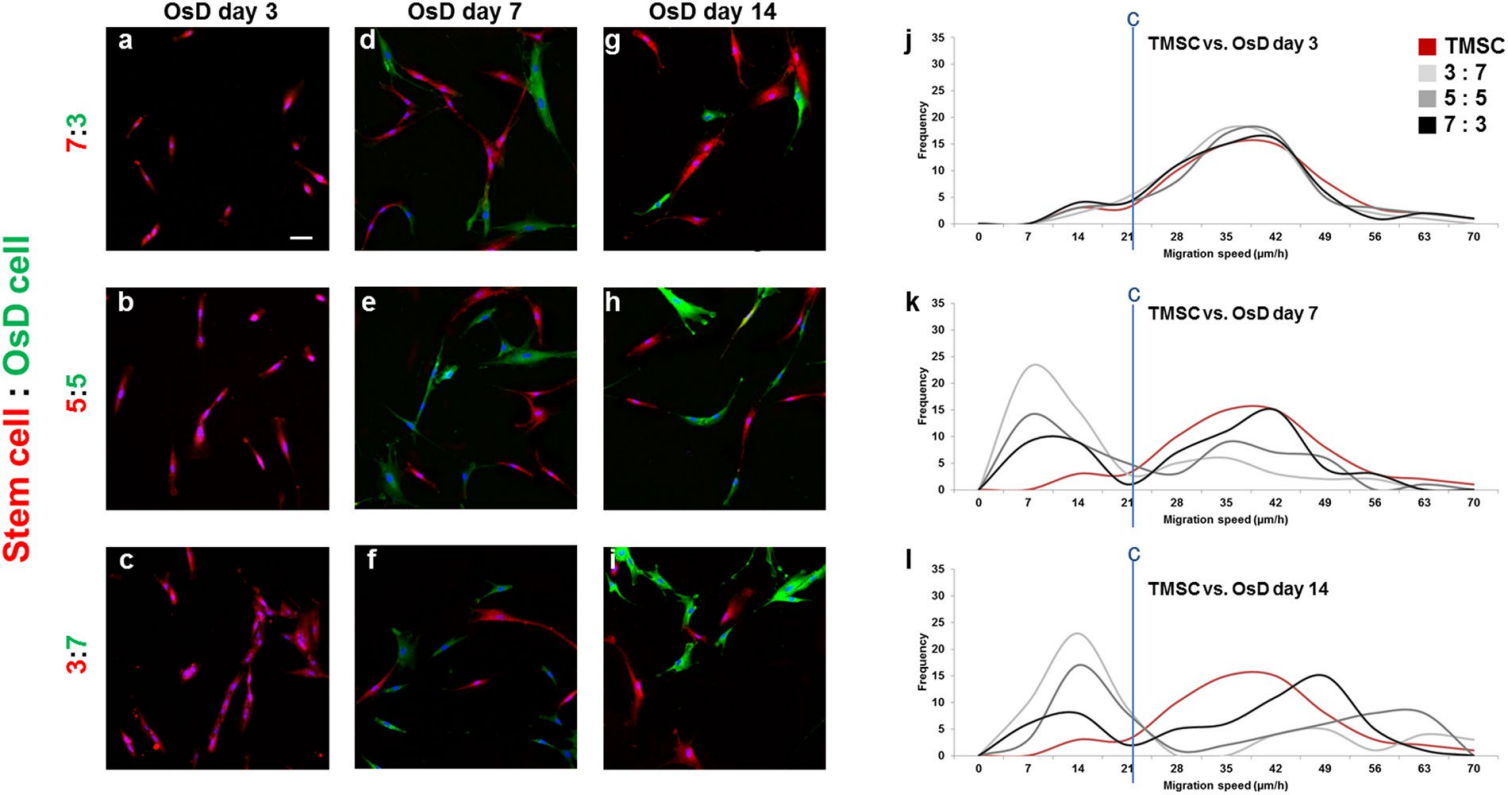
Mixture of TMSC and osteogenic differentiated cells at different OsD days. The immunofluorescence images of TMSC and osteogenic differentiated cells mixture ratio of (**a**) 7:3, (**b**) 5:5, (**c**) 3:7 at day 3, (**d**) 7:3, (**e**) 5:5, (**f**) 3:7 at day 7, and (**g**) 7:3, (**h**) 5:5, (**i**) 3:7 at day 14. The histogram of TMSC and osteogenic differentiated cell mixture using electrotaxis analysis and Youden index under 1000 µA at (**g**) day 3, (**h**) day 7, (**i**) day 14. Cut point of TMSC vs. osteogenic differentiated cells was 22.42 μm/h. Scale bar = 100 μm.

**Figure 7:**
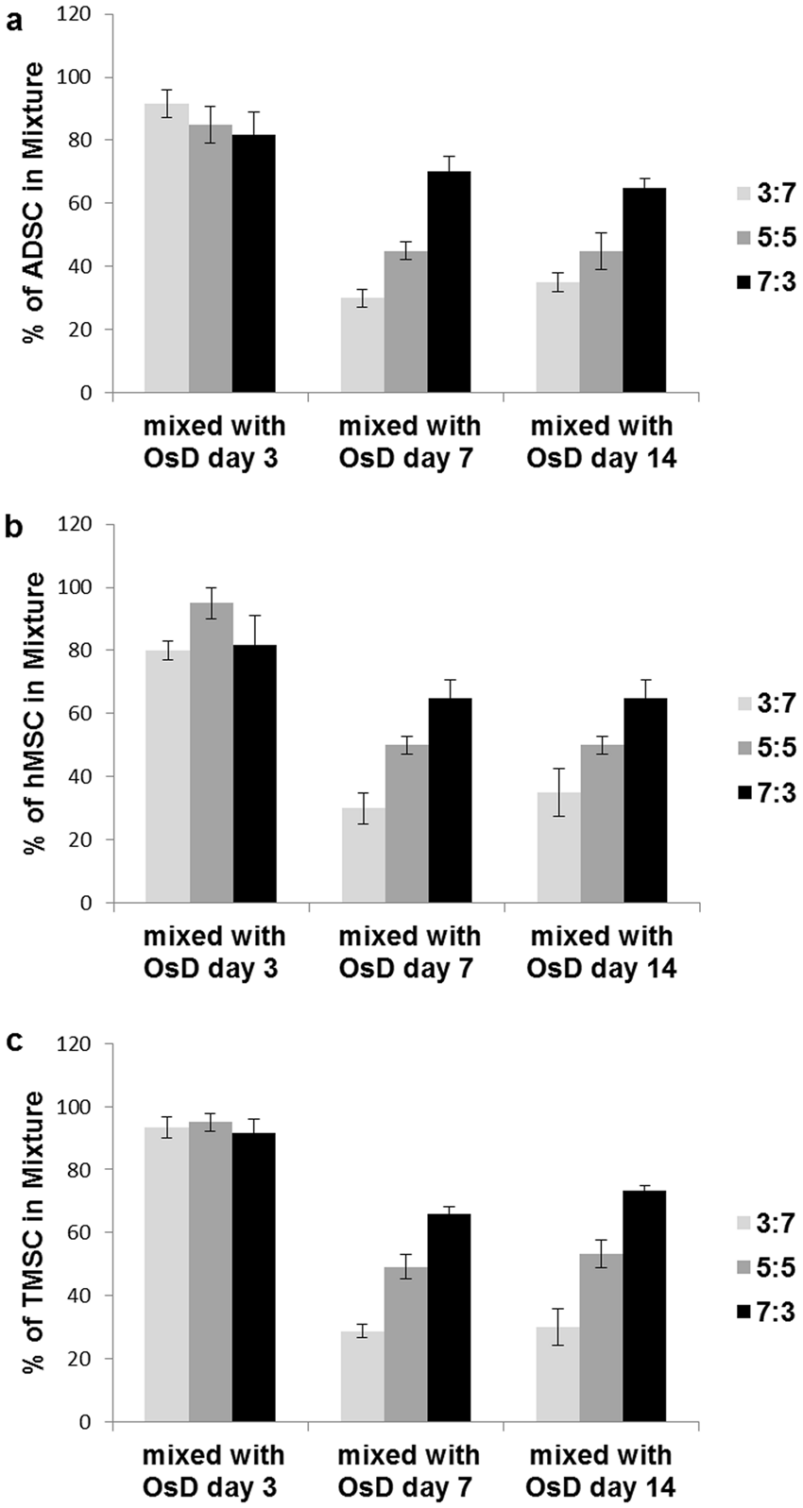
Homogeneity evaluation of mesenchymal stem cells based on electrotaxis analysis. (**a**) Evaluation data of ADSC homogeneity in mixture of ADSC and osteogenic differentiated cells. (**b**) Evaluation data of hMSC homogeneity in mixture of hMSC and osteogenic differentiated cells. (**c**) Evaluation data of TMSC homogeneity in mixture of TMSC and osteogenic differentiated cells.

To evaluate the homogeneity of stem cells, the mixtures of stem cells and osteogenic differentiated cells was prepared; ADSC vs. osteogenic differentiated cell, hMSC vs. osteogenic differentiated cell, TMSC vs. osteogenic differentiated cell. Each mixture group has 3 different ratios and the ratios were 7:3, 5:5, 3:7. We identify the homogeneity of stem cells using the stem cell marker and osteogenic differentiation maker, then these homogeneity data by markers were compared with homogeneity data by electrotaxis analysis. Figure 4(a)~(i) showed the fluorescence images of ADSC and osteogenic differentiated cell population at OsD day 3, 7, 14. The population of ADSC could be identified at OsD day 7 and day 14 by OsD marker and stem cell marker (Fig. 4(d)~(i)). Before evaluate the ADSC homogeneity by electrotaxis analysis, the migration speed histogram of ADSC and osteogenic differentiated cell mixture was performed first. The frequency of ADSC and ratio 3:7, 5:5, 7:3 was almost matched at OsD day 3 (Fig. 4j). However, the frequency of ADSC and ratio 3:7, 5:5, 7:3 has a difference at OsD day 7, day 14 (Fig. 4k,l). These results mean that the heterogeneity of mixture at 3d could not be detected by fluorescence images or electrotaxis analysis, however the population of ADSC and osteogenic differentiated cells was possible to be detected by RUNX2 and CD-105 marker, and electrotaxis analysis at day 7, day 14 (Fig. 4d~l). The same analysis was applied to hMSC and TMSC mixture, too. The population of hMSC could be identified at OsD day 7 and day 14 by OsD marker and stem cell marker, but not be identified at OsD day 3 (Fig. 5a~i). The migration speed histogram of hMSC and osteogenic differentiated cell mixture showed that the frequency of hMSC and ratio 3:7, 5:5, 7:3 was almost matched at OsD 3d (Fig. 5j). However, the frequency of hMSC and ratio 3:7, 5:5, 7:3 has a difference at OsD day 7, day 14 (Fig. 5k,l). In TMSC and OsD mixture, similar data were also obtained (Fig. 6). Finally we evaluate the homogeneity of MSCs by electrotaxis and Youden index analysis (Fig. 7). In mixture of ADSC and OsD day 3 cells, the percentage of ADSC was 91.7 ± 4.4% at 3:7 ratio, 85.0 ± 5.8% at 5:5 ratio, 81.7 ± 7.3% at 7:3 ratio. In mixture of ADSC and OsD day 7 cells, the percentage of ADSC was 30.0 ± 2.9% at 3:7 ratio, 45.0 ± 2.9% at 5:5 ratio, 70.0 ± 5.0% at 7:3 ratio. In mixture of ADSC and OsD day 14 cells, the percentage of ADSC was 35.0 ± 2.9% at 3:7 ratio, 45.0 ± 5.8% at 5:5 ratio, 65.0 ± 2.9% at 7:3 ratio (Fig. 7(a)). In mixture of hMSC and OsD day 3 cells, the percentage of hMSC was 80.0 ± 2.9% at 3:7 ratio, 95.0 ± 5.0% at 5:5 ratio, 81.7 ± 9.3% at 7:3 ratio. In mixture of hMSC and OsD day 7 cells, the percentage of hMSC was 30.0 ± 5.0% at 3:7 ratio, 50.0 ± 2.9% at 5:5 ratio, 65.0 ± 5.8% at 7:3 ratio. In mixture of hMSC and OsD day 14 cells, the percentage of hMSC was 35.0 ± 7.6% at 3:7 ratio, 50.0 ± 2.9% at 5:5 ratio, 65.0 ± 5.8% at 7:3 ratio (Fig. 7(b)). In mixture of TMSC and OsD day 3 cells, the percentage of TMSC was 93.3 ± 3.3% at 3:7 ratio, 95.0 ± 2.9% at 5:5 ratio, 91.7 ± 4.4% at 7:3 ratio. In mixture of TMSC and OsD day 7 cells, the percentage of TMSC was 28.9 ± 2.0% at 3:7 ratio, 49.2 ± 4.0% at 5:5 ratio, 66.0 ± 2.0% at 7:3 ratio. In mixture of TMSC and OsD day 14 cells, the percentage of TMSC was 30.0 ± 58% at 3:7 ratio, 53.3 ± 4.4% at 5:5 ratio, 73.3 ± 1.7% at 7:3 ratio (Fig. 7(c)). The percentage of MSCs in mixture with OsD cells was almost matched with each ratio, so these results suggested that the possibility about the evaluation of MSCs homogeneity by electrotaxis analysis was identified.

## Discussion

The heterogeneity of stem cell therapy products hampers not only the fast translation of research into clinics but also the proper interpretation of clinical trial data. This heterogeneity could originate from intrinsically heterogeneous initial cell populations and additional diversity of cell populations during the stem cell culture process^7^. Thus, as important as it is to implement a standardized stem cell culture process, evaluation technique of stem cell homogeneity to reduce the heterogeneity is critical to stem cell therapy.

There are some studies that hypoxic environment promoted the osteogenic differentiation of stem cells^22, 23^. Other reports demonstrated that human bone marrow-derived MSCs cultured under hypoxia showed a diminished capacity to differentiate into adipocytes and osteocytes, supporting the notion that low oxygen tension promotes an undifferentiated state^24–26^. Especially the current differentiation protocols lead to unsynchronized the birth and development of cell populations and different differentiation stages^4–7^. According to these studies, stem cell quality control is very important when we culture the stem cells in stem cell differentiation.

Direct current electric fields are present naturally at wounds in a variety of different tissues and are a powerful directional guidance cue for epithelial cells, fibroblasts, vascular endothelial cells, keratinocytes, endothelial progenitor cells, and neurons. Directed migration of cells in a DC EF is highly cell-type specific, since some cell types migrate cathodically and others anodically^27^. Here, 3 types of MSCs showed strong anodal electrotaxis in physiological electric current (Fig. 2). However, mouse adipose-derived stromal cells (mASC) migrated towards the cathode in response to EFs^28^. In our hands, osteogenic differentiated cells from ADSC, hMSC and TMSC migrated anodally (Fig. 2). This difference in electrotaxis could be related to differences in cell types, tissue sources and species, thereby emphasising the need for comprehensive studies of the effects of EF application on a case-by-case basis. The underlying mechanisms of MSC electrotaxis for each cell type need to be clarified, nevertheless it is believed that the electrotaxis is the specificity of each cells. The direct current induced directional migration of hMSC but did not effect on osteogenic differentiation^26^, so our electrotaxis analysis technique which enables the real time observation of cell characteristics during cell cultures is a candidate for stem cell quality control method.

For some stem cell types, the initial cell populations can be isolated from various tissues or organs, and depending on how strict the isolation selection criteria are, some heterogeneity can be introduced by this choice of source. For example, MSCs have been sourced from bone marrow, adipose tissue (AT), umbilical cord blood, umbilical cord tissue, and tonsil^12, 29^. Comparative studies showed AT and umbilical cord blood-sourced MSCs showed higher colony frequency and better proliferation capacity, respectively, compared with BM-MSCs^30^. Phenotypically, ADSCs express CD34, whereas BM-derived MSCs do not^31^. Because of these different characteristics from stem cell types, stem cells isolated from various tissues (bone marrow, adipose tissue, tonsil) were selected in this study for identifying the evaluation technique base on the electrotaxis analysis as a wide use to stem cell homogeneity evaluation.

In Fig. 1, all 3 types of stem cells, hMSC, ADSC and TMSC showed the expression of RUNX2 which represented the osteogenic differentiation marker at day 7, 14. Figure 2 showed that migration speed of all 3 types of differentiated cells was significantly decreased at OsD day 7, 14 however the electric current had no effect on the migration speed. These results suggested that the electric current effected on only the directional migration of stem cells and 0 differentiated cells, so electrotaxis analysis has the possibility of using to the stem cell homogeneity evaluation.

For stem cell quality control by electrotaxis, it is very important that the electric treatment should not affect to differentiation and viability of stem cells. For these reason, post-electrotaxis experiments were performed. We identified that there was no effect of 1000 µA of electric current on the osteogenic differentiation of ADSC, hMSC, TMSC (S1–S3). The viability of stem cells after 1000 µA of electric current was also checked and as a result, stem cell viability was not affected by 1000 µA of electric current (Fig. 3). These data strongly assured that electrotaxis is good candidate for safe method of stem cell quality control.

To distinguish the stem cell and differentiated cells, Youden index of migration speed under electrotaxis was used. Generally Youden index is used to evaluate biomarker levels in the investigation and diagnosis of disease. Disease diagnosis by biomarkers is dependent upon a correlation between biomarker levels and disease state, whereby biomarker levels for a certain diseased population are different–usually higher–than in the corresponding non-diseased population^32, 33^. Figure 2 showed the more osteogenic differentiation progressed, the more migration-speed decreased so stem cell homogeneity is dependent upon a correlation between migration speed and osteogenic differentiation state. In order to utilize migration speed for such classification, a cut-point is established and individuals with migration speed values on one side of the cut-point are labeled as stem cells and those with values on the other side are labeled osteogenic differentiated cells.

Figures 4–6 showed that the mixture ratio 7:3, 5:5, 3:7 of stem cells and OsD cells was matched with the homogeneity evaluation data based on electrotaxis analysis. In OsD 3d, it was hard to distinguish the stem cells and OsD 3d cells by fluorescence markers because only CD-105 was detected and RUNX2 was not (Figs 4(a)~(c)–6(a)~(c)). Similarly, the electrotaxis analysis data showed no different patterns between stem cells and OsD 3d (Figs 4(j)–6 (j)). However, stem cells showed red dyes (CD-105) and OsD 7, 14d cells showed green dyes (RUNX2) so the distinguishing of stem cells and OsD cells was possible (Figs 4(d)~(i)–6(d)~(i)). The electrotaxis analysis data also showed different pattern between stem cells and OsD 7, 14d cells that the more OsD cells increased, the more low migration speed cells increased too (Figs 4(k,l)–6(k,l)). Figure 7 showed that the percentage of stem cells which was calculated using cut-off value was matched with the mixture ratio. These results suggested that OsD took time at least more than 3 days and stem cells moved slower when they started to become OsD cells. This is the key characteristic for stem cell quality control based on the electrotaxis analysis.

According to all results in this study, our stem cell homogeneity technique base on electrotaxis analysis evaluated the stem cell population in culture condition without any specific antibodies so this technique might be used as the stem cell quality control during stem cell culture. Also stem cells which were isolated from various tissues showed common results with this technique, so our evaluation tool could be the wide use to stem cell homogeneity evaluation.

## Conclusions

Our study demonstrates the possibility about evaluation of stem cell population by the electrotaxis analysis. The mixture ratio of MSCs and osteogenic differentiated cells was almost matched with the distinguishment of MSCs by the electrotaxis analysis. Our study emphasizes the importance of electric current in stem cell migration analysis because the presence of the electric current made more accurate result of discrimination between MSCs and osteogenic differentiated cells. Our result might be used as the new method to evaluate the homogeneity of stem cells.

## Electronic supplementary material

Supplementary Information
