# Author Correction: Homogeneity evaluation of mesenchymal stem cells based on electrotaxis analysis

**DOI:** 10.1038/s41598-018-31445-y

**Published:** 2018-10-25

**Authors:** Min Sung Kim, Mi Hee Lee, Byeong-Ju Kwon, Dohyun Kim, Min-Ah Koo, Gyeung Mi Seon, Jong-Chul Park

**Affiliations:** 1 Cellbiocontrol Laboratory, Department of Medical Engineering, Yonsei University College of Medicine, Seoul, 120-752 Korea; 2 Brain Korea 21 PLUS Project for Medical Science, Yonsei University College of Medicine, Seoul, 120-752 Korea

Correction to: *Scientific Reports* 10.1038/s41598-017-09543-0, published online 29 August 2017

The Acknowledgements section in this Article is incorrect.

“This research was supported by the Bio & Medical Technology Development Program of the National Research Foundation (NRF) funded by the Korean government (MEST) (No. 2015M3A9E2028643).”

should read:

“This research was supported by the National Research Foundation of Korea (NRF), funded by the Ministry of Science, ICT & Future Planning (Grant No. 2012–049729)”.

