# Supplementary material for: Homogeneity evaluation of mesenchymal stem cells based on electrotaxis analysis
Source: Sci Rep. 2017 Aug 29;7:9582. doi: 10.1038/s41598-017-09543-0 (PMC5574884; doi:10.1038/s41598-017-09543-0)
Supplement: Supplementary file 1 — Supplementary Information [file 41598_2017_9543_MOESM1_ESM.pdf]

# Homogeneity evaluation of mesenchymal stem cells based on electrotaxis analysis

MIN SUNG KIM,[a,b] MI HEE LEE,[a] BYEONG-JU KWON,[a,b] DOHYUN KIM,[a] MIN-AH KOO,[a,b] GYEUNG MI SEON,[a,b] JONG-CHUL PARK[a,b,*]

[a]Cellbiocontrol Laboratory, Department of Medical Engineering, Yonsei University College of Medicine, Seoul 120-752, Korea

[b]Brain Korea 21 PLUS Project for Medical Science, Yonsei University College of Medicine, Seoul 120-752, Korea

*Correspondence should be addressed to: Cellbiocontrol Laboratory, Department of Medical Engineering, Yonsei University College of Medicine, 134 Shinchon-dong, Seodaemun-gu, Seoul 120-752, Korea. Tel: +82 2 2228 1917. Fax: +82 2 363 9923. Electronic mail: parkjc@yuhs.ac

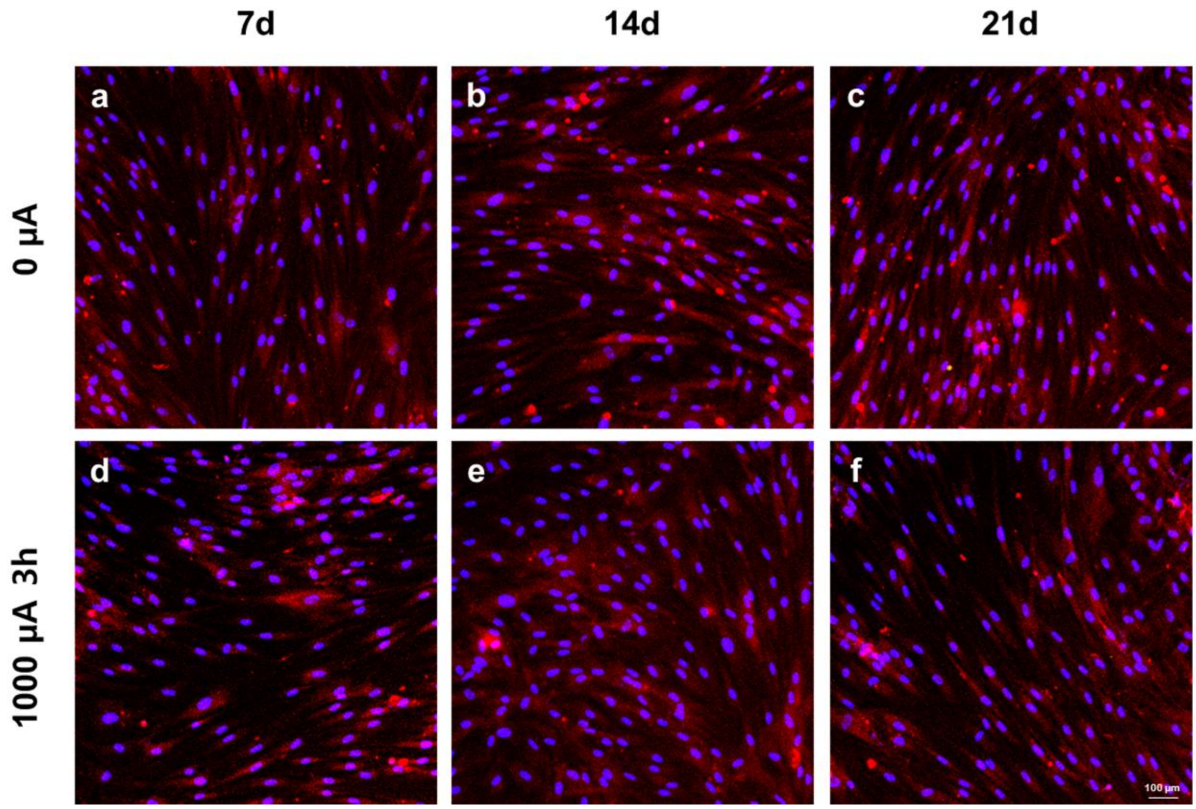


Supplementary figure S1. The effect of direct electric current on ADSC osteogenic differentiation. The fluorescence images of ADSC cultured in ADSCGM at (a) 7d, (b) 14d, (c) 21d without electric treatment. The fluorescence images of ADSC cultured in ADSCGM at (a) 7d, (b) 14d, (c) 21d after 1000 μA of 3h electric treatment (nuclei: Hoechst, blue / stem cell marker: CD-105, red / osteogenic differentiation marker: RUNX2, green). Scale bar = 100 μm.

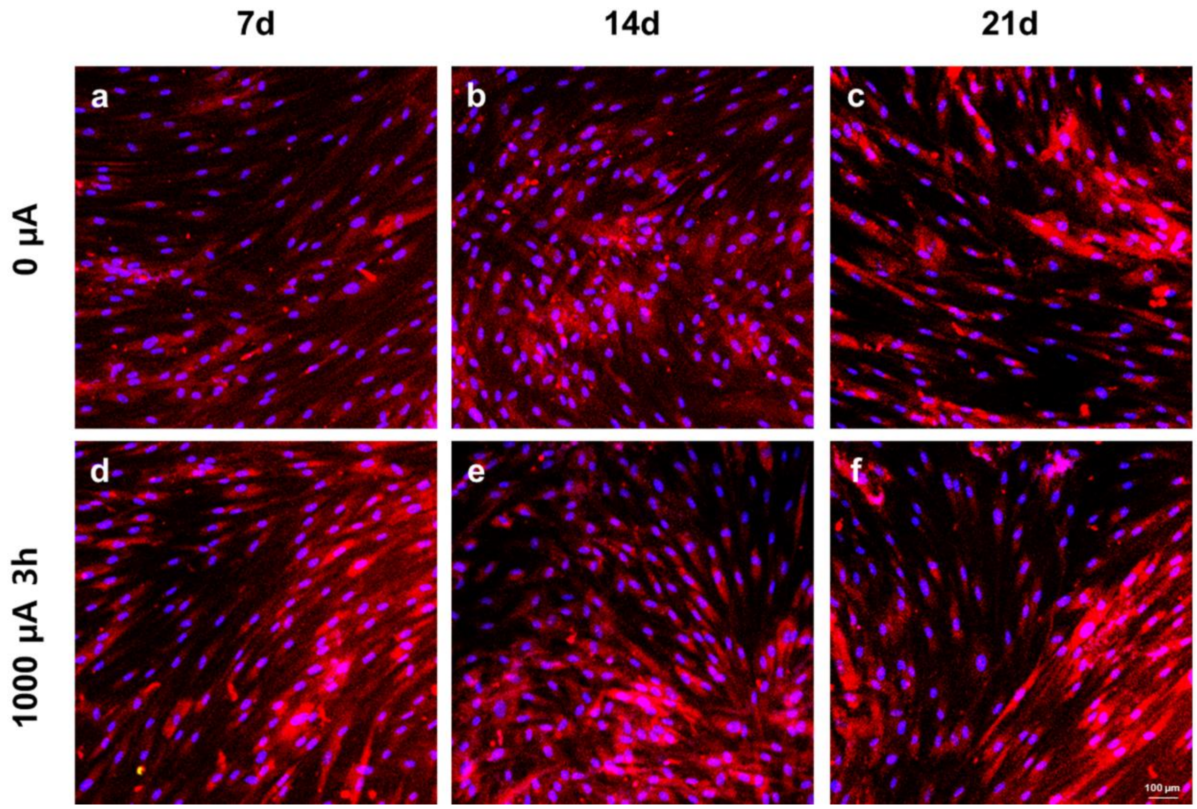


Supplementary figure S2. The effect of direct electric current on hMSC osteogenic differentiation. The fluorescence images of hMSC cultured in MSCGM at (a) 7d, (b) 14d, (c) 21d without electric treatment. The fluorescence images of ADSC cultured in MSCGM at (a) 7d, (b) 14d, (c) 21d after 1000 μA of 3h electric treatment (nuclei: Hoechst, blue / stem cell marker: CD-105, red / osteogenic differentiation marker: RUNX2, green). Scale bar = 100 μm.

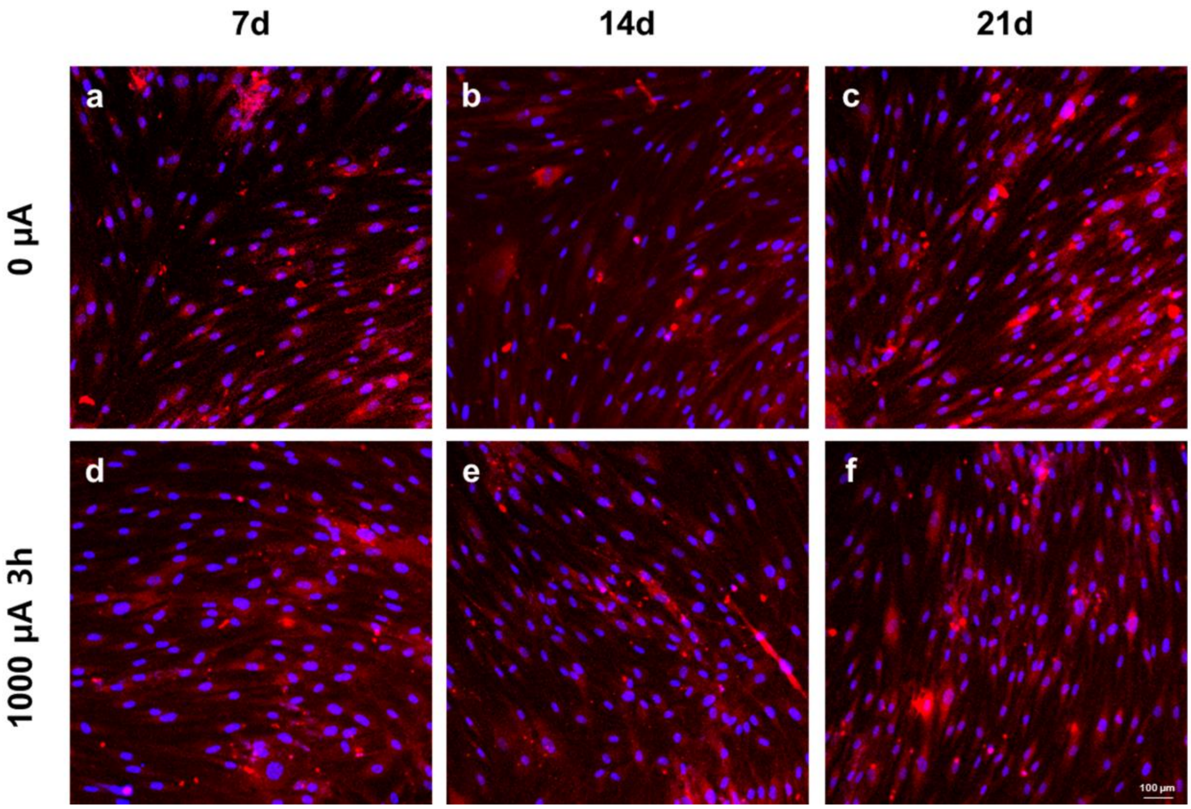


Supplementary figure S3. The effect of direct electric current on TMSC osteogenic differentiation. The fluorescence images of TMSC cultured in DMEM at (a) 7d, (b) 14d, (c) 21d without electric treatment. The fluorescence images of TMSC cultured in DMEM at (a) 7d, (b) 14d, (c) 21d after 1000 μA of 3h electric treatment (nuclei: Hoechst, blue / stem cell marker: CD-105, red / osteogenic differentiation marker: RUNX2, green). Scale bar = 100 μm.
